# Supplementary material for: Identification of a Rice Leaf Width Gene Narrow Leaf 22 (NAL22) through Genome-Wide Association Study and Gene Editing Technology
Source: Int J Mol Sci. 2023 Feb 17;24(4):4073. doi: 10.3390/ijms24044073 (PMC9962836; doi:10.3390/ijms24044073)
Supplement: Supplementary file 1 [file ijms-24-04073-s001.zip › Short legends for Supporting Information.pdf]

Figure S1. Expression pattern in different tissues of 77 genes in LALW4 and LALW5. The X-axis represents different tissues in different growth stages. The Y-axis represents the RAP-DB ID of genes. Different degrees of red represent different log_2(FPKM) values. Red marked genes represent two candidate genes.

Figure S2. Sequence analysis of *CG1-CG6* in rice accessions with extremely wide and narrow LW. The gene number is in the upper left corner. "ATG-" represents upstream of "ATG"; "ATG+" represents exon and intron; "TGA+, TAA+, and TAG+" represent downstream of the stop codon. "*" represents the nonsynonymous mutation; "!" represents the synonymous mutation. The table below the positions shows the specific sequence variations. "A, T, G and C" in the table are the bases. "+" and "-" indicate insertions and deletions. "I^1- I^6" represent the insertion of "ATACATTAAA", "TACAGAG", "GTCTCGTCCC", "AGTCTCTGGATCCAAACACC", "ATTTT", and "AGAGAG", respectively. "D^1" represents deletion of "GCGCGCGCGC".

Figure S3. Number of large veins, small veins and small veins in adjacent large veins. (A) The number of large veins. (B) The number of small veins. (C) Number of small veins in adjacent large veins. Gray bars indicate ZH11; red bars indicate *nal22-2*, purple bars indicate *nal22-3*. Black bars indicate the difference between two *nal22* mutants. "ns" represents no significant difference.

Figure S4. Transcription level of genes related to cell elongation. (A) The transcription level of *EXPA10*. (B) Transcription of *EXPB5*. (C) Transcription of *GH9B1*. Gray bars indicate ZH11; red bars indicate *nal22-2*, purple bars indicate *nal22-3*. "**" represents significant differences ($P<0.01$), and "ns" represents no significant difference.

Figure S5. Root phenotype of *nal22* mutants. (A) Phenotype of roots of ZH11 and *nal22* mutants. (B) Bar diagram of root length. Gray bars indicate ZH11; red bars indicate *nal22-2*, purple bars indicate *nal22-3*. Black bars indicate the difference between two *nal22* mutants. "*" represents significant differences ($P<0.05$), and "ns" represents no significant difference.

Figure S6. Phylogenetic relationship and conserved motif composition of 78 *homologous* proteins of *NAL22*. A phylogenetic tree was constructed based on the amino acid sequences, calculated by the neighbor-joining method with 1,000 bootstrap replicates and created by MEGA 5. Numbers are the bootstrap values. "MafL" indicates "Maf-like protein"; "m7GTP" indicates 7-methyl-GTP pyrophosphatase-like protein; "HyPr" indicates hypothetical protein. Schematic diagram of the conserved motif homologous proteins predicted by MEME. Colored boxes indicate different motifs. The red marked protein is *NAL22*.

Figure S7. Transcription level of *NAL22* in the *gid1* mutant under water and GA_3 treatments at different time points. The concentration of GA_3 was 100 micromoles. "ns" represents no significant difference.

Figure S8. Sequence alignment of NAL22 and two subfamilies of Maf proteins. Six sequences above NAL22 were in the YhdE subfamily. Five sequences below NAL22 were in the YceF subfamily. Residues conserved in all aligned proteins are highlighted in blue. The signature residues for the YhdE and YceF subfamilies are highlighted in green and orange, respectively. The residues conserved in the YhdE and YceF subfamilies and the different residues in NAL22 are highlighted in yellow and red, respectively. The abbreviation corresponding species name: EC-*E. coli,* BS-*B. subtilis*, PF-*Pyrococcus furiosus*, Mbar-*Methanosarcina barkeri*, SC-*S. cerevisiae*, HS-human, STM-*Salmonella typhimurium*, Dd-*Dictyostelium dsicoideum*, PA-*Pseudomonas aeruginosa*, and Tb-*T. brucei.*

Figure S9. Predicted protein structure of NAL22 and comparison with YhdE. (A) Protein structure and active enzyme sites of YhdE. (B) Predicted protein structure and active enzyme sites of NAL22. Red arrows mark the difference in the cavity in YhdE and NAL22.

Table S1. Primers used in this paper.

Table S2. Leaf width of different rice accessions in field.

Table S3. The 12 LALWs and their rice genomic information.

Table S4. Reported QTLs associated with leaf width.

Table S5. List of genes in LALW4 and LALW5.

Table S6. Expression profile of candidate genes in different tissues.

Table S7. *cis*-elements in the promoter of *NAL22*.
