# Supplementary figures and images for: Identification of a Rice Leaf Width Gene Narrow Leaf 22 (NAL22) through Genome-Wide Association Study and Gene Editing Technology
Source: Int J Mol Sci. 2023 Feb 17;24(4):4073. doi: 10.3390/ijms24044073 (PMC9962836; doi:10.3390/ijms24044073)

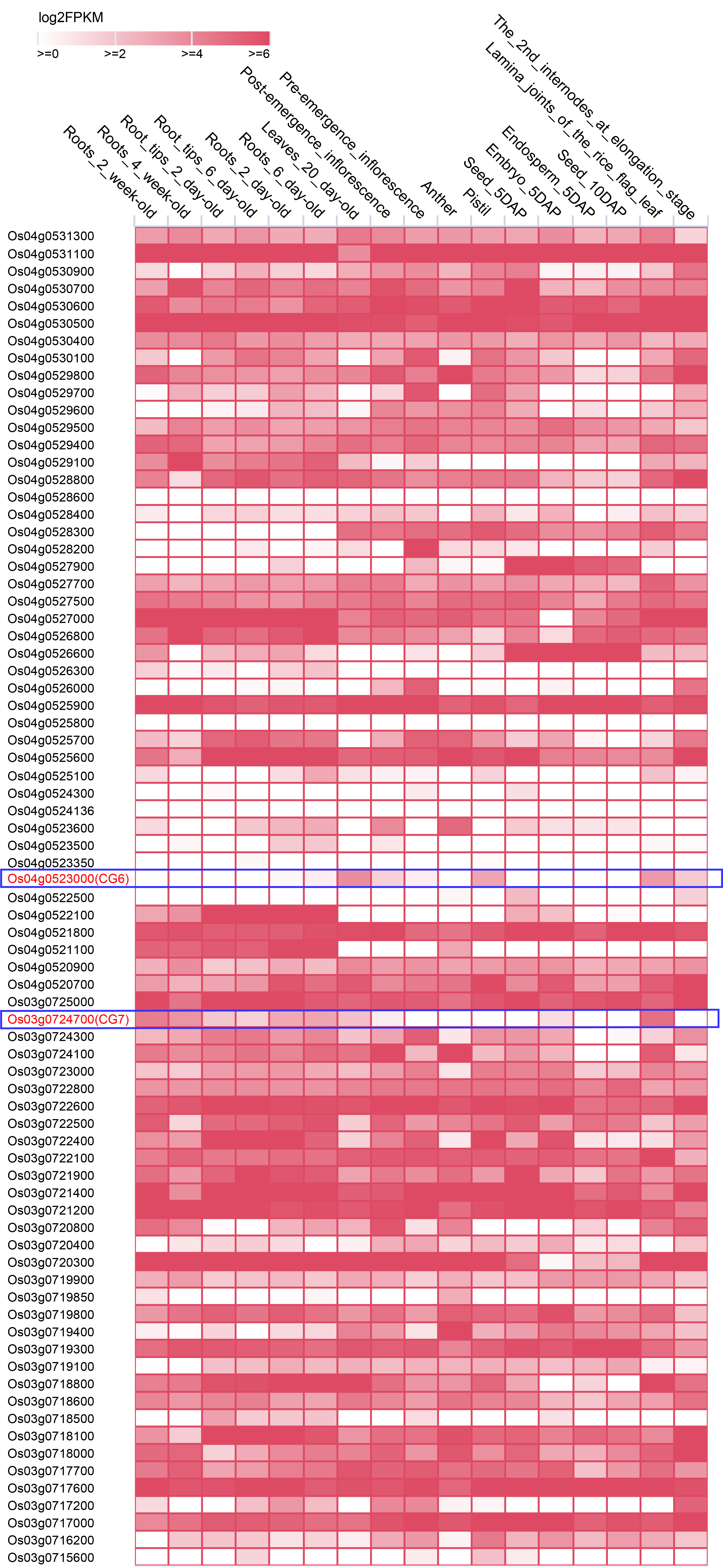

Supplement: Supplementary file 1 [file ijms-24-04073-s001.zip › Supplementary figure S1.jpg]

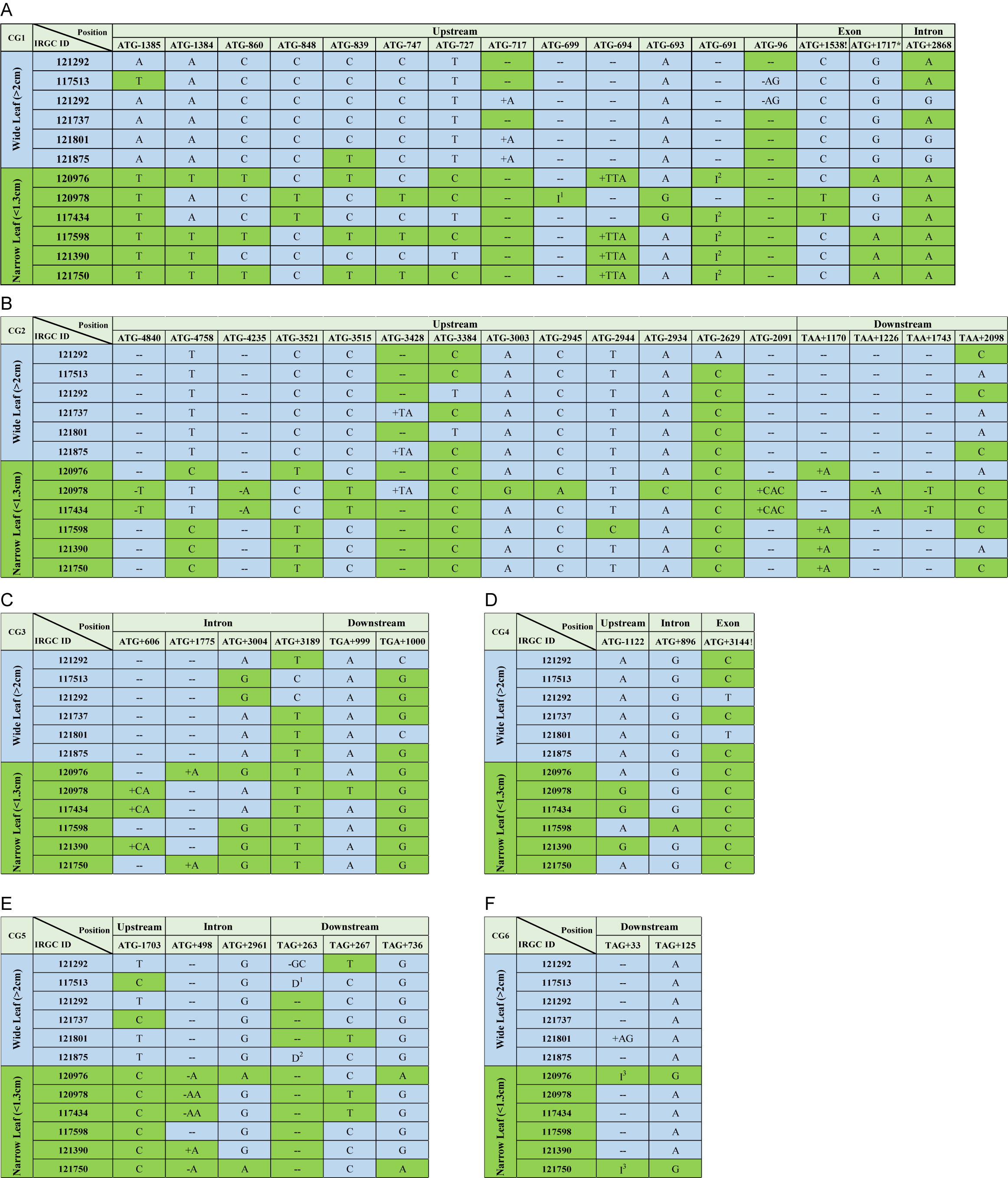

Supplement: Supplementary file 1 [file ijms-24-04073-s001.zip › supplementary figure S2.jpg]

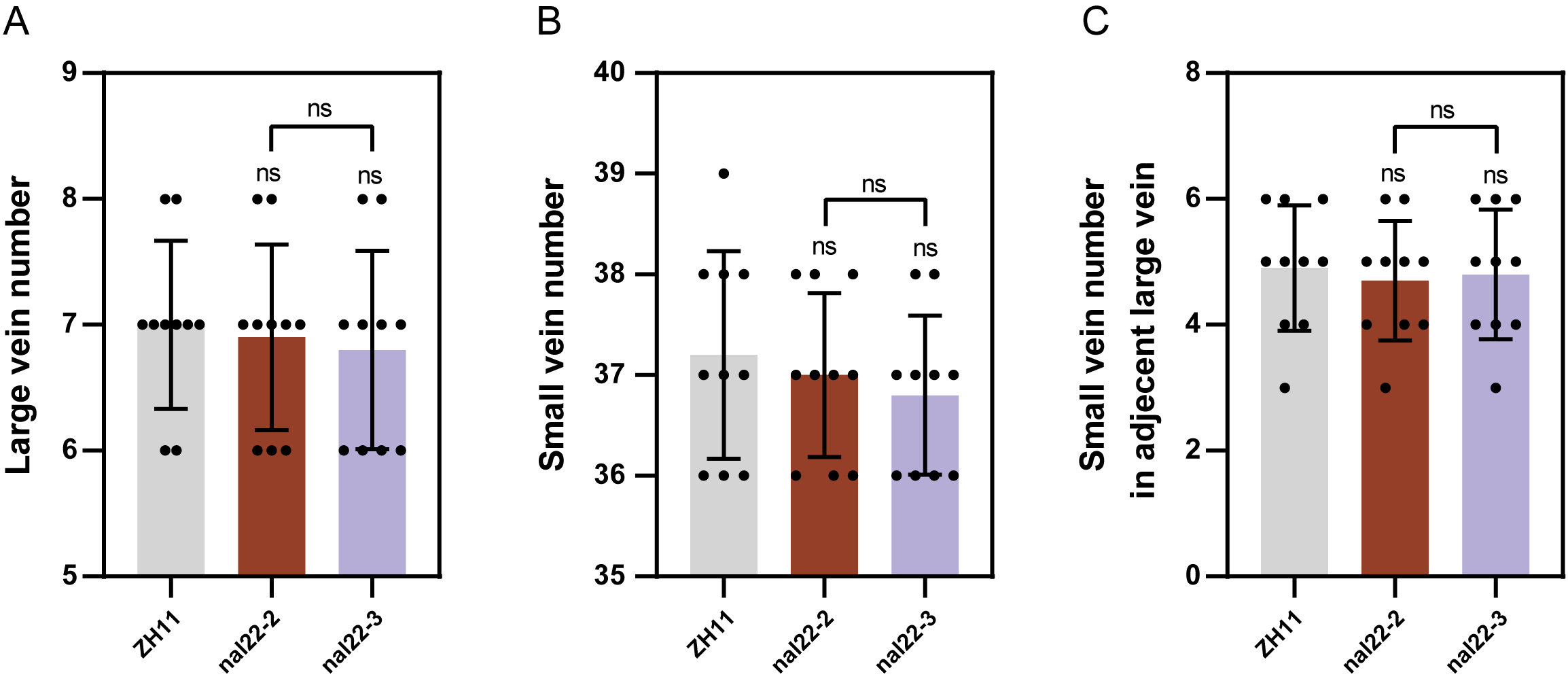

Supplement: Supplementary file 1 [file ijms-24-04073-s001.zip › supplementary figure S3.jpg]

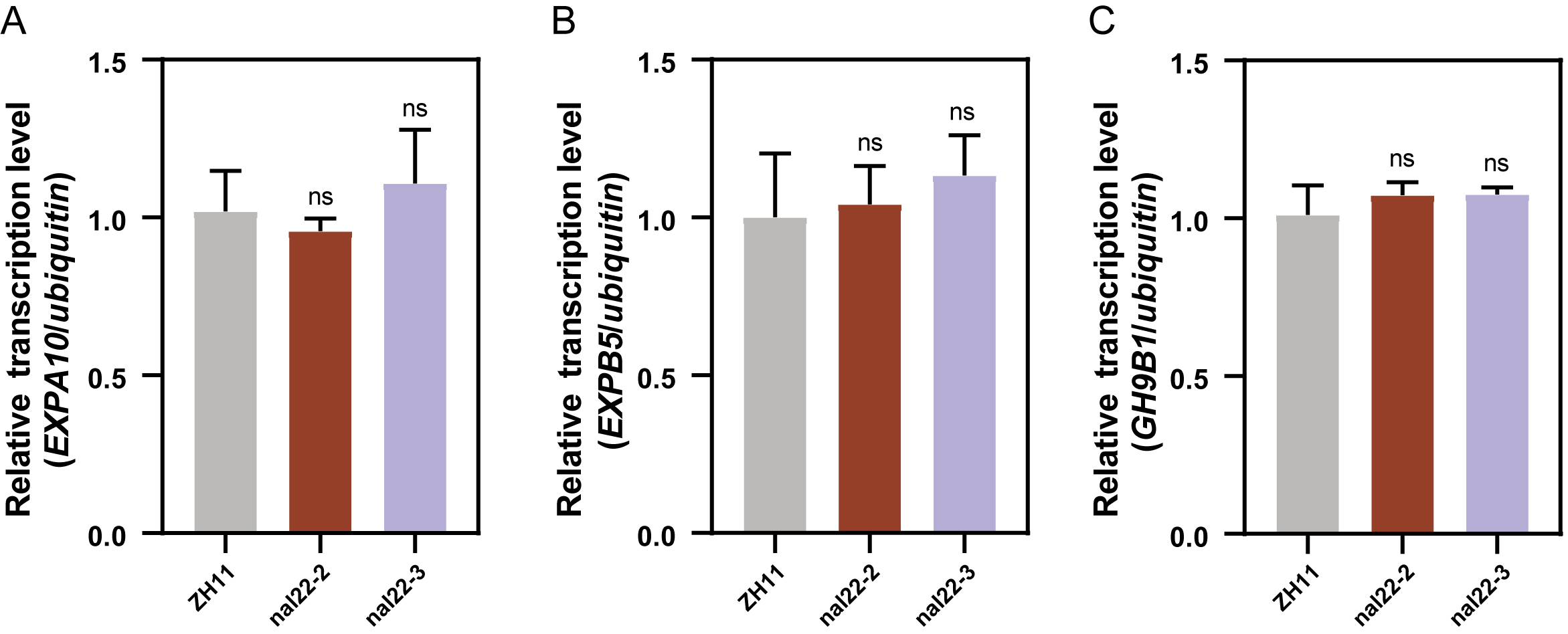

Supplement: Supplementary file 1 [file ijms-24-04073-s001.zip › supplementary figure S4.jpg]

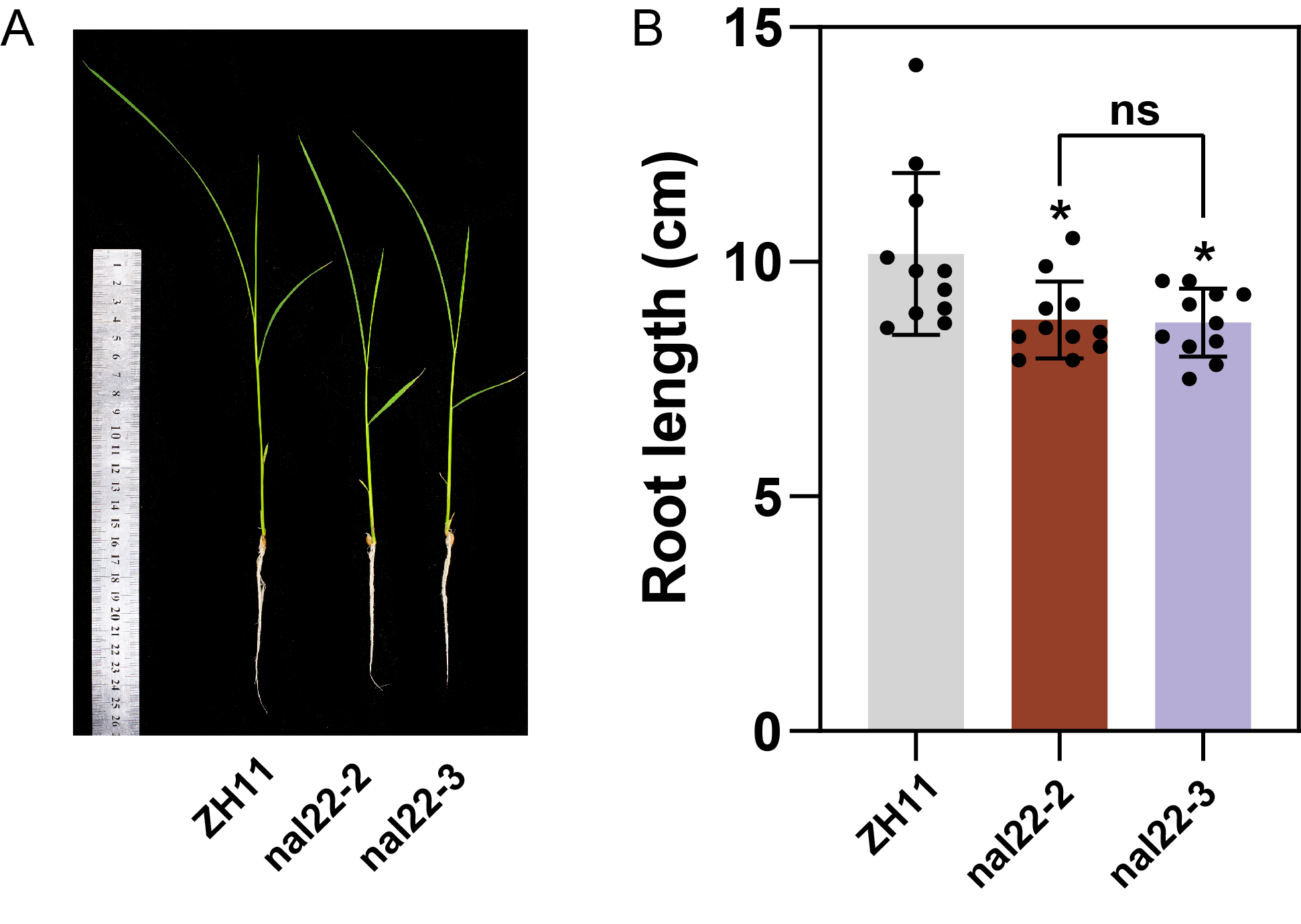

Supplement: Supplementary file 1 [file ijms-24-04073-s001.zip › supplementary figure S5.jpg.jpg]

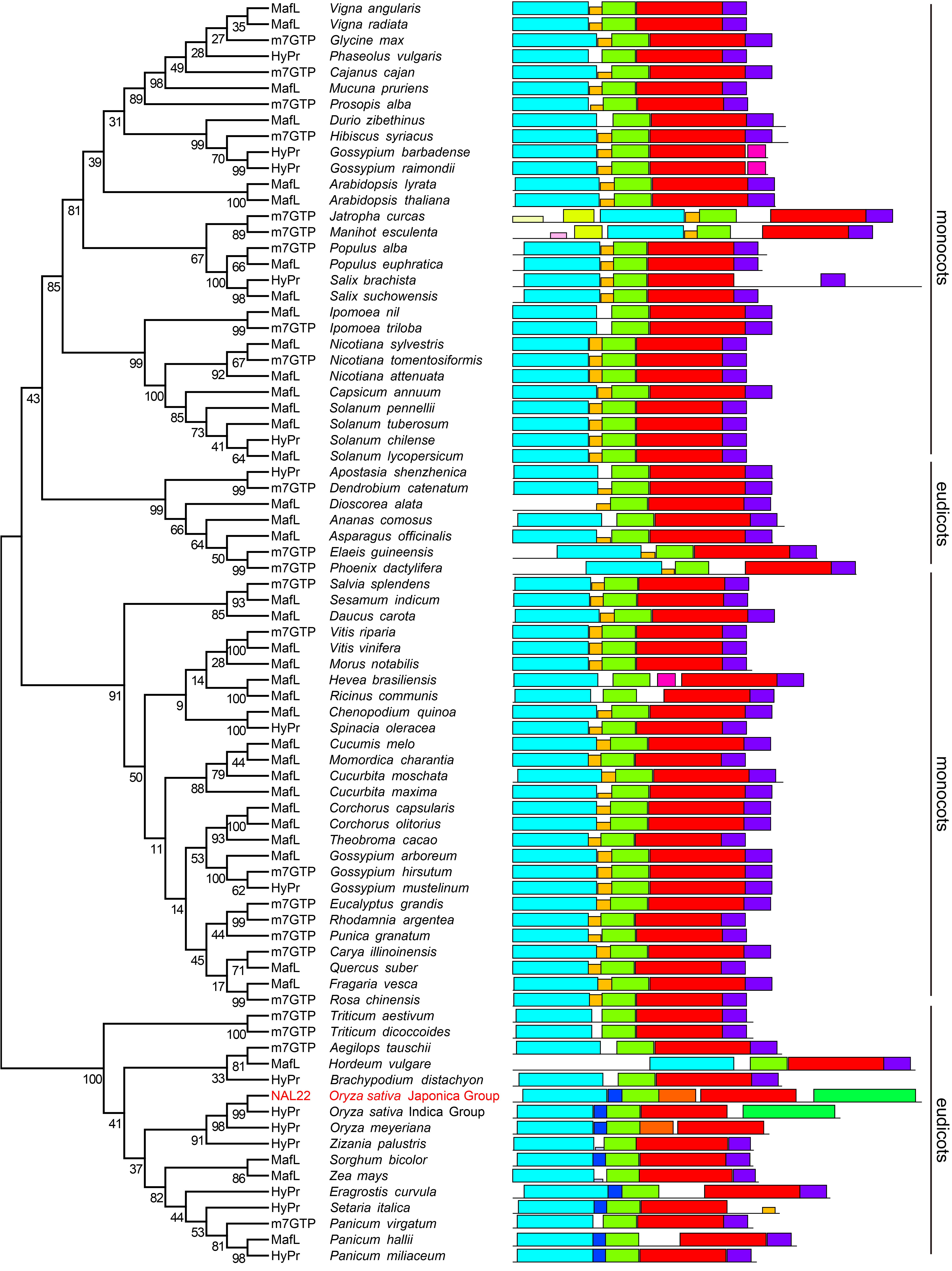

Supplement: Supplementary file 1 [file ijms-24-04073-s001.zip › Supplementary figure S6.jpg]

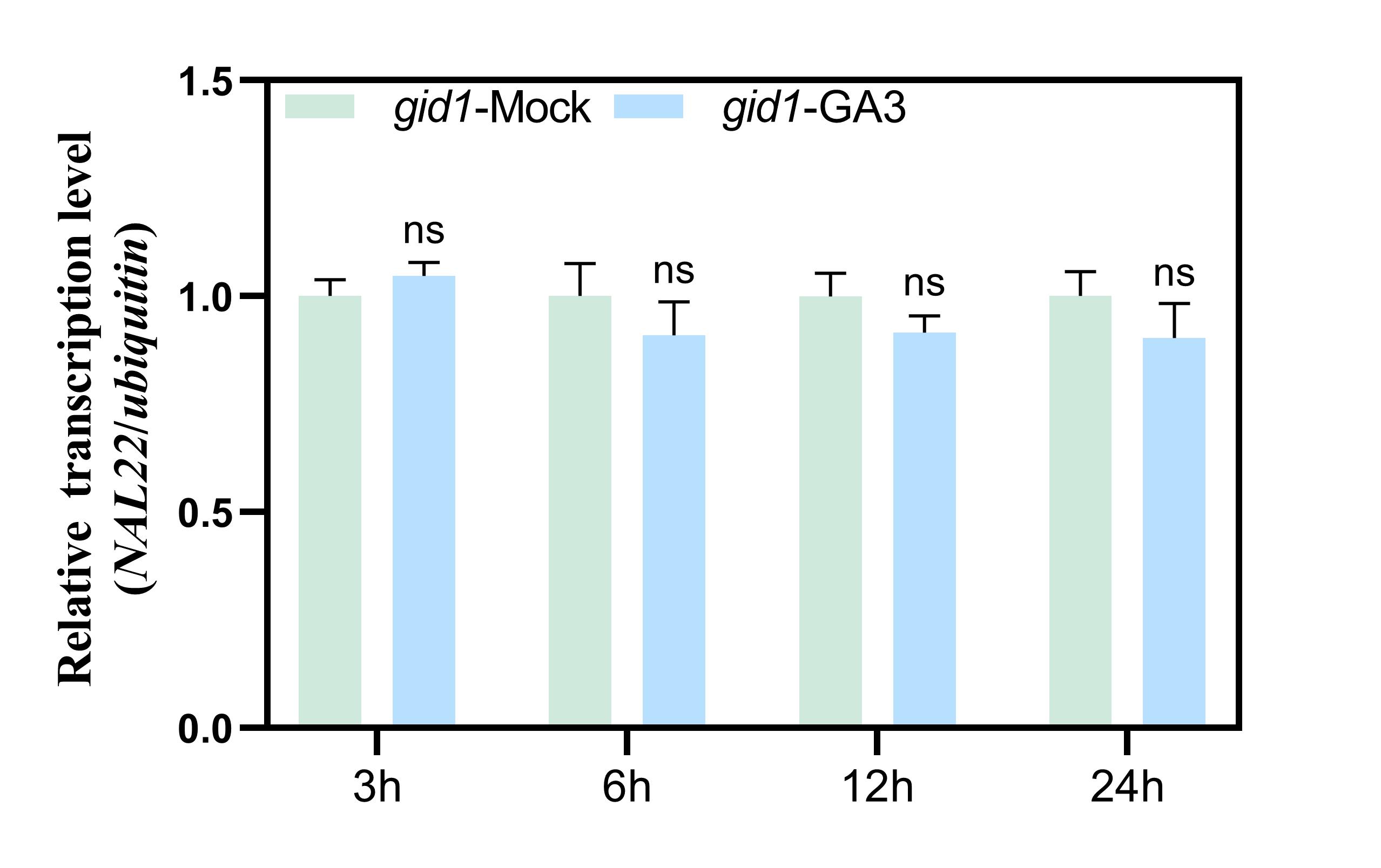

Supplement: Supplementary file 1 [file ijms-24-04073-s001.zip › Supplementary figure S7.jpg]

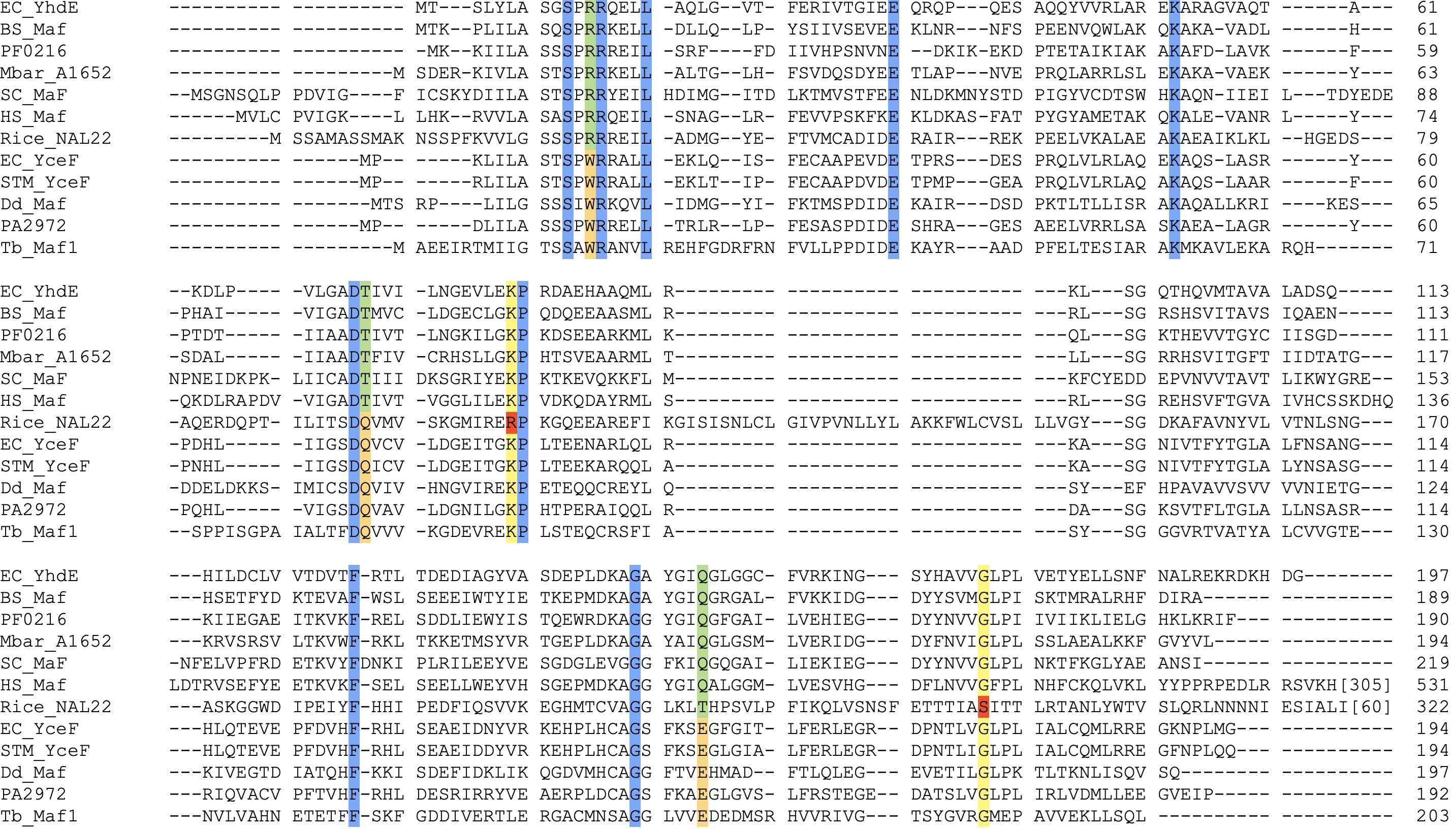

Supplement: Supplementary file 1 [file ijms-24-04073-s001.zip › Supplementary figure S8.jpg]

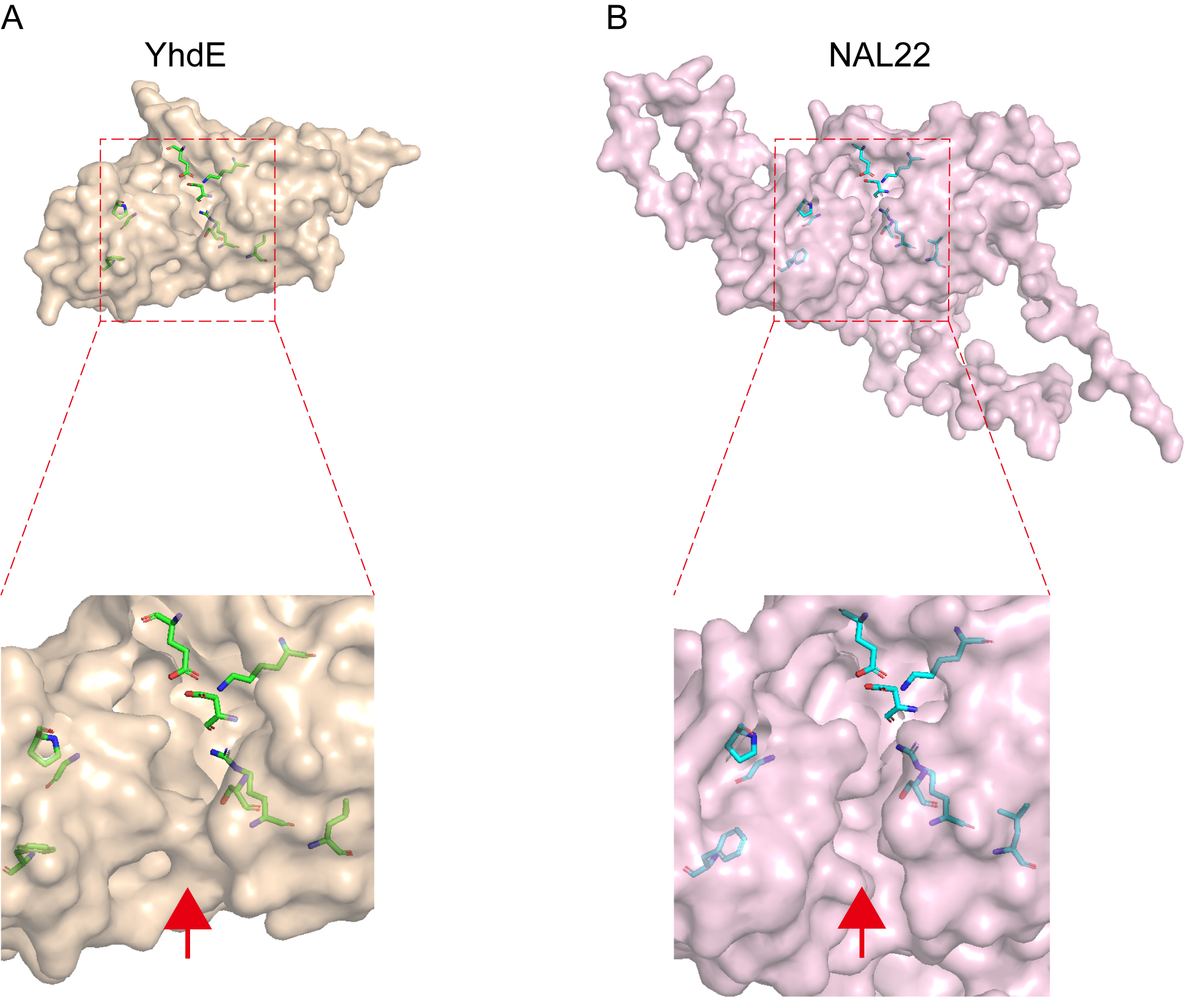

Supplement: Supplementary file 1 [file ijms-24-04073-s001.zip › supplementary figure S9.jpg]
